# Longitudinal changes of lipid profile in the Lebanese pediatric population

**DOI:** 10.1186/s12944-019-0991-x

**Published:** 2019-02-11

**Authors:** Chloé Saadé, Ghassan Sleilaty, Marie-Hélène Gannagé-Yared

**Affiliations:** 1 Endocrinology Department, Faculty of Medicine, Saint Joseph University, Saint-Joseph, Beirut, Lebanon; 2 Biostatistics Department, Faculty of Medicine, Saint Joseph University, Saint-Joseph, Beirut, Lebanon; 3 Division of Endocrinology, Hotel-Dieu de France Hospital, Beirut, Lebanon

**Keywords:** Longitudinal, Lipid, Lebanese, School-age children

## Abstract

**Background:**

Few studies looked at the prevalence of dyslipidemia in pediatric Middle-Eastern countries. In addition, worldwide longitudinal changes of lipid profile is not well documented. The purpose of this study is to look at the longitudinal changes of lipid parameters in Lebanese school-age children.

**Materials and methods:**

A total of 97 subjects (41 girls and 56 boys) aged between 11 and 21 years were included in this study. The subjects were selected among 339 school-age children with a previous abnormal lipid profile who were recruited from 10 schools of varying socio-economic levels (SEL). A fasting lipid profile [total cholesterol (TC), triglycerides (TG) and HDL-cholesterol (HDL-C)] was performed. Non-HDL-cholesterol (Non-HDL-C) was calculated. Weight and height were measured under the same conditions, and BMI percentiles were calculated. A multivariate covariance analysis model (MANCOVA) was used with TG, HDL-C and non-HDL-C as dependent variables with additional post-MANCOVA F tests.

**Results:**

The age of the current cohort is 16.5 ± 2.9 years with no significant difference according to gender. The current lipid profile was obtained 3.1 ± 0.7 years following the initial one, with 53.6% of the subjects having it normalized. TC, TG, and non-HDL-C decreased significantly over time in girls, while only TG decreased significantly in boys. No significant changes were observed for HDL-C. Using MANCOVA, a significant time by age interaction was observed (*p <* 0.0001), while gender, BMI and SEL were found not to be significant. Post-hoc F tests showed that the time by age interaction was driven by TG (*p* = 0.03) and non-HDL-C (*p* < 0.001), the larger effect being observed in younger children.

**Conclusion:**

A high proportion of school-age children normalize their abnormal lipid profile with time. Screening for lipid disorders could be postponed until post puberty age.

## Introduction

Disorders of lipoprotein metabolism are major cardiovascular risk factors in addition to smoking, high blood pressure and type 2 diabetes, leading to atherosclerosis and cardiovascular diseases [1–3]. There is mounting evidence that atherosclerosis begins early in childhood. Subsequently, identification of dyslipidemia during childhood and adolescence would prevent the progression of atherosclerosis [4]. Traditionally, screening for dyslipidemia in children targeted only individuals at-risk with a family history of familial hypercholesterolemia. Since 2011, new recommendations have introduced universal screening for dyslipidemia to reduce the risk of cardiovascular disease beginning in childhood [5], but currently, there is a lack of evidence for benefits of universal screening versus selective screening, making the first one controversial [6, 7].

In the Middle East region, metabolic syndrome, diabetes mellitus, familial hypercholesterolemia and consanguinity are highly prevalent and result in a variety of lipid disorders: low High Density Lipoprotein-Cholesterol (HDL-C), high triglycerides (TG) and high Low Density Lipoprotein-Cholesterol (LDL-C) [8]. In Lebanon, the few studies that looked at the prevalence of dyslipidemia were mainly cross-sectional and carried out on adult populations [9, 10]. These studies were performed using either a single-cholesterol measurement [9] or were done on a selected subgroup of population such as hospital employees [10]. Only one cross-sectional study was carried out on a pediatric population by one of the authors of the current paper [MHG] [11], showing that the prevalence of high non-HDL-C and TG were respectively 9.2 and 26.6% with significant variations by gender, body mass index (BMI) and socioeconomic level (SEL).

Other cross-sectional studies have described changes of lipid levels by age during childhood and adolescence, in a similar way to the previous Lebanese report [11], with a tendency toward worsening of lipid profile in boys and improvement in girls after puberty [4, 12–14]. These studies were performed in US [7, 12–14], Europe [15, 16] and in the Middle-East [17], but longitudinal studies are sparse [18, 19].

Since there are no longitudinal studies of dyslipidemia in Lebanon, the purpose of this study is: 1) to assess the longitudinal changes of lipid profile in children with a previous abnormal lipid profile; 2) to assess the factors that might affect these variations, mainly age, gender, BMI and SEL.

## Methods

### Population

This is a longitudinal study performed on a sample of the Lebanese school-age children population recruited during 2013/2014 from a previously studied pediatric population [11] in 10 schools from the Great Beirut and Mount Lebanon areas. Schools were classified by socio-economic level (SEL), depending on the annual school fees. The current sample was selected among the 339 subjects of the previous study who have showed an abnormal lipid profile and who agreed to participate in this second part of the study. Recruitment period extended from August 2016 until October 2017.

The exclusion criteria were any acute or chronic illness (such as diabetes and hypothyroidism), any medical treatment that may affect the lipid profile (lipid lowering drugs, contraceptive pill, isotretinoin, corticosteroids, atypical antipsychotics and immunosuppressants) and lack of signed informed consent (of parents for minor children or currently adult subjects).

In the present study, all the parents or legal representatives of the children with an abnormal lipid status in the 2013/2014 cohort were contacted. Results of the first lipid profile were sent back to schools’ physicians for appropriate follow-up. Only children with familial hypercholesterolemia were excluded from this study: they were directly contacted by our group to implement treatment. None of the included subjects reported to follow a lipid lowering diet.

Informed consent was signed by the parents of minor children and the adult participants. The project was approved by the Ethics Committee of the Hôtel-Dieu de France hospital (CEHDF, tfem-2017-74).

### Measurements

Weight and height of all the participants were measured under the same conditions. The BMI was calculated according to the formula: weight / (height)^2^ where weight was expressed in kilograms and height in meters. Normal weight was defined by a BMI <85th percentile, overweight by a BMI between the 85th and 95th percentile, and obesity by a BMI ≥ 95th percentile. The 2000 Centers for Disease Control and Prevention (CDC) curves that apply for subjects aged between 2 and 20 years were used to calculate the percentiles of BMI in the absence of normative curves in the Lebanese population [20]. In the current sample, 2 subjects were 21 years old, consequently they were considered to be 20 years old for BMI percentiles calculation purposes.

### Biological analysis

Blood samples were taken after twelve hours of fasting and were analyzed on the same day, using a Hitachi 912 automate, in the biochemistry laboratory of our institution. The blood analysis included TC, HDL-C and TG. Non-HDL-C was calculated by subtracting HDL-C from TC. Abnormal values were defined according to the recommendations of the NHLBI as follows: 1) in subjects younger than 20 years old (TC > 5.2 mmol / L (200 mg / dL), LDL-C > 3.4 mmol / L (130 mg / dL), HDL-C < 1 mmol / L (40 mg / dL), non-HDL-C > 3.8 mmol / L (145 mg / dL) and TG either > 1.29 mmol/L (100 mg/dL) in children younger than 9 years old or > 1.46 mmol/L (130 mg/dL) in subjects between 10 and 19 years old); 2) in young adults aged between 20 and 24 years old (TC > 5.8 mmol / L (225 mg / dL), LDL-C > 4.1 mmol / L (160 mg / dL), HDL-C < 1 mmol / L (40 mg / dL), non-HDL-C > 4.9 mmol / L (190 mg / dL) and TG > 1.69 mmol/L (150 mg/dL)).

### Statistical analysis

To rule out selection biases, univariate statistics including T test and Chi square test were used to compare the 2013/2014 values of the 97 subjects included in the present study with those of the overall cohort showing an abnormal lipid profile (*n* = 339). The observed changes in lipid profile between 2013/2014 and 2016/2017 were compared using a paired T-test.

A multivariate analysis of covariance (MANCOVA) was performed using a linear combination of the lipid parameters (HDL-C, TG, Non-HDL-C) as the dependent variable. A multivariate Wilk test was used to assess the effect of interaction on the dependent variable due to time, gender, age, BMI percentiles and SEL. Post hoc univariate F tests were performed to analyze separately each component of the dependent variable. The adequacy of the model was based on Cook distances and the studentized ranges.

Data were analyzed using the SPSS software (IBM Corp. Released 2013. IBM SPSS Statistics for Windows, Version 22.0.Armonk, NY: IBM Corp.)

## Results

### Comparison of the previous values (2013/2014) of the current sample with those of the entire initial cohort

Among the 339 subjects with abnormal lipid profile in 2013/2014, 97 subjects agreed to participate again in the current phase and their characteristics in 2013/2014 were compared to those of the 339 subjects included in the initial cohort. No significant differences were found between the 2 groups other than in SEL (Table 1).

**Table 1:** Comparison of baseline characteristics (2013/2014) of the current sample (*n* = 97) with characteristics of the entire sample who had dyslipidemia in 2013/2014 (*n* = 339)

	2013/2014	2016/2017	*P* value
	cohort	sub-cohort	
	(*N* = 339)	(*n* = 97)	
Boys/Girls (n)	175/164	56/41	0.29
Age (yrs)	13.4 ± 2.9	13.5 ± 3.3	0.84
SEL			
Low	180	31	<0.0001
Middle	92	35	
High	67	31	
BMI percentiles	71.1 ± 28.1	69.3 ± 27.5	0.57
Total Cholesterol (mmol/l)	4.41 ± 0.96	4.52 ± 0.85	0.32
Triglycerides (mmol/l)	1.93 ± 0.79	1.85 ± 0.83	0.45
HDL-Cholesterol (mmol/l)	1.1 ± 0.28	1.15 ± 0.33	0.30
Non-HDL-Cholesterol (mmol/l)	3.31 ± 0.91	3.37 ± 0.75	0.50

Age corresponds to the age of subjects in 2013/2014

### Current characteristics of the sample

Out of the 97 subjects included in the follow-up sample, 56 were males (57.7%) and 41 were females (42.3%), with an average age of 13.4 ± 3.3 years in the first sampling and 16.5 ± 2.9 years in the second one. The mean time interval between the two samples is 3.1 ± 0.7 years in the entire sample (3.0 ± 0.8 years for males and 3.3 ± 0.7 years for females, *p* = 0.58). None of the subjects reported to follow. All the included subjects and/or their legal representative assured that there had been no change in their diet/lifestyle at the time of the second sampling, despite their having the results of the first sampling.

### Longitudinal changes in lipid parameters

The values of BMI percentiles and of the different components of the lipid profile (TC, HDL-C, Non-HDL-C, TG) in the entire sample and by gender during the 2 study periods (2013/2014, 2016/2017) are depicted in Table 2. Out the 97 subjects, 45 subjects (46.4% of the sample) still show at least one abnormal value in the lipid profile. In the total population, a significant decrease of TC, TG, and non-HDL-C (*p* = 0.02, *p* < 0.0001 and *p* < 0.0001 respectively) was noted, while there was no significant change in BMI percentiles and in HDL-C. In girls, we observed a significant decrease in the TC, non-HDL-C and TG (*p* = 0.001, *p* < 0.0001 and *p* < 0.0001 respectively) while the values did not change for HDL-C and BMI percentiles. Finally, in boys, we observed only a significant decrease of TG (*p* < 0.0001).

**Table 2:** Longitudinal changes in the lipid profile in the entire sample and by gender

	Boys	Girls	Total
	*n* = 56	*n* = 41	*n* = 97
BMI percentiles 1	69.46 ± 28.74	69.03 ± 25.04	69.28 ± 27.10
BMI percentiles 2	68.63 ± 25.26	63.83 ± 25.41	66.60 ± 25.30
* P*-value	0.69	0.06	0.1
TC1	4.37 ± 0.80	4.72 ± 0.88	4.52 ± 0.85
TC2	4.22 ± 0.77	4.42 ± 0.83	4.30 ± 0.80
* P*-value	0.13	0.001	0.02
HDL-C1	1.11 ± 0.29	1.19 ± 0.38	1.15 ± 0.33
HDL-C2	1.12 ± 0.30	1.27 ± 0.43	1.19 ± 0.37
* P*-value	0.76	0.08	0.13
Non-HDL-C1	3.25 ± 0.77	3.53 ± 0.72	3.37 ± 0.75
Non-HDL-C2	3.09 ± 0.77	3.15 ± 0.64	3.12 ± 0.72
* P*-value	0.06	<0.0001	<0.0001
TG1	1.92 ± 0.84	1.76 ± 0.81	1.85 ± 0.83
TG2	1.25 ± 0.65	1.23 ± 0.52	1.24 ± 0.60
* P*-value	<0.0001	<0.0001	<0.0001

TC1, HDL-C 1, Non-HDL-C 1, TG 1 and BMI1 Percentiles are the values in the 2013/2014 cohort

TC2, HDL-C 2, Non-HDL-C 2, TG 2 and BMI2 Percentiles are the values in the 2016/2017 cohort

Lipid profile values expressed in mmoL/L

*p*-values correspond to the results of paired T tests that compare the 2016/2017 results with the 2013/2014 results in the entire sample and by gender

### Effect of gender, BMI, SEL on the change of lipid profile (Table 3)

**Fig. 1:**
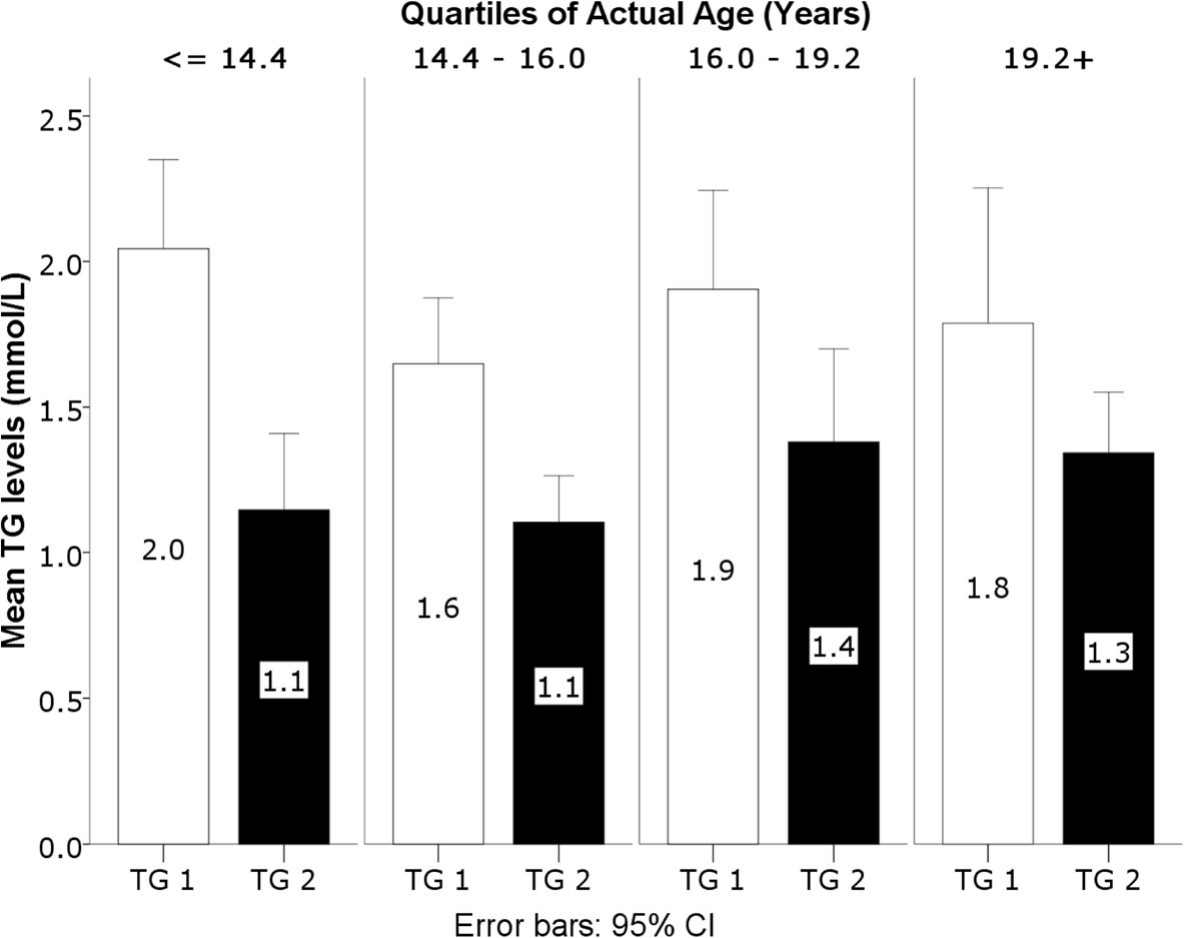
Interaction between TG levels and age**.** TG are expressed in mmol/L at the time of the original assessment in 2013/2014 (TG 1) and the follow-up assessment in 2016/2017 (TG 2). TG levels are shown by quartiles of actual age

**Fig. 2:**
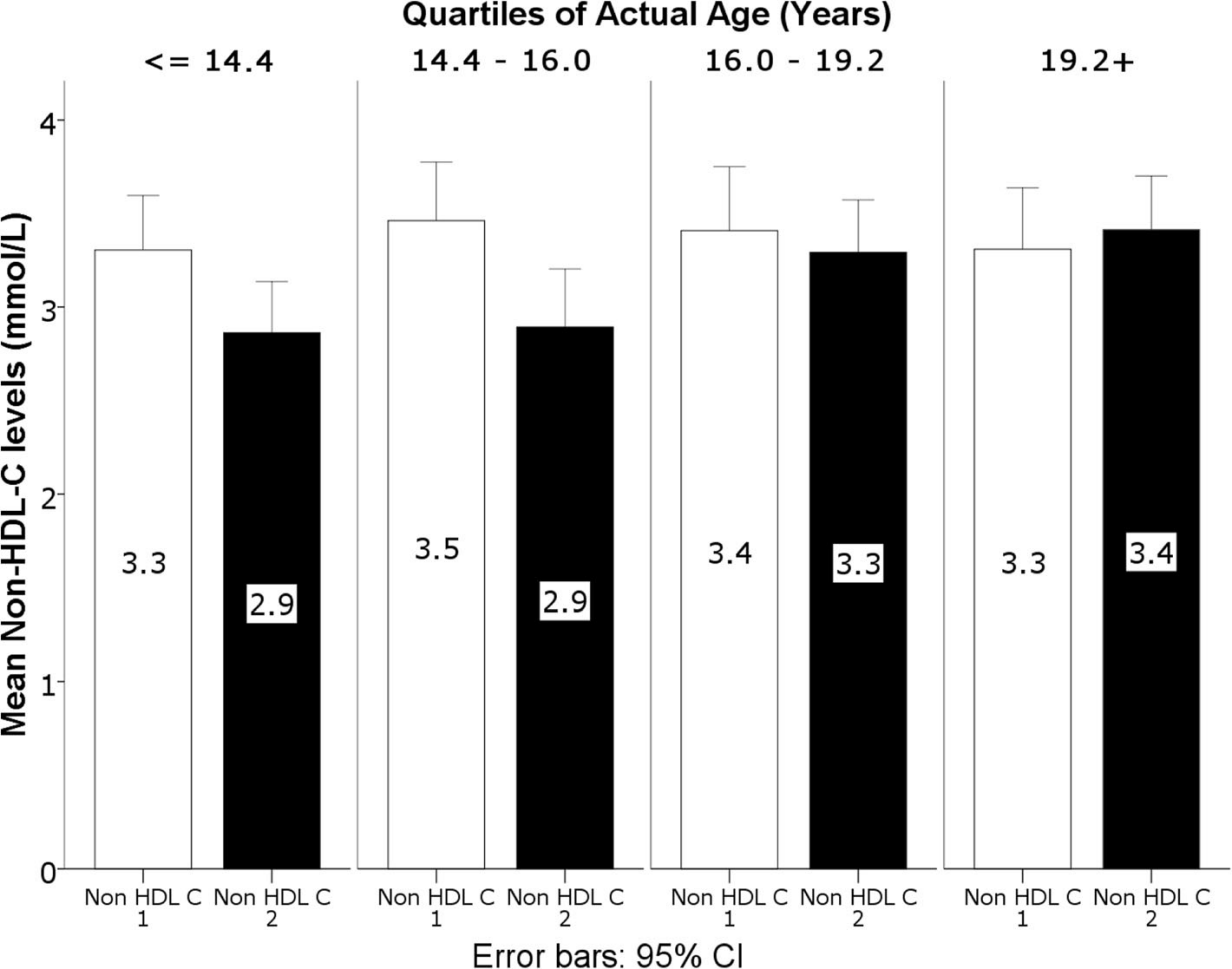
Interaction between Non-HDL-C levels and age**.** Non-HDL-C are expressed in mmol/L at the time of the original assessment in 2013/2014 (Non-HDL-C 1) and the follow-up assessment in 2016/2017 (Non-HDL-C 2). Non-HDL-C levels are shown by quartiles of actual age

MANCOVA was used to assess the variability of the linear combination of non-HDL-C, TG and HDL-C (Table 3). A significant effect of time was observed (*p* = 0.001), and most importantly a significant time by age interaction was found (*p* < 0.0001), while the interaction effects of BMI percentiles, gender and SEL did not reach statistical significance (*p* = 0.50, *p* = 0.09 and *p* = 0.89 respectively). Post MANCOVA F tests showed that the time effect and time by age interaction were driven by TG (*p* = 0.009 for time effect, *p* = 0.028 for time by age interaction) and non-HDL-C (*p* = 0.001 for time effect, *p* < 0.0001 for time by age interaction). The time by age interaction resulted from a farther decrease of TG and non-HDL-C in younger children as depicted in Figs. 1 and 2 (first and second quartiles).

**Table 3:** Results of the Univariate F tests following MANCOVA, showing the time by factor interaction, with the corresponding *p*-values, effect sizes and observed power

Source	Parameter	*p*-value	Effect size (Partial Eta Squared)	Observed Power^a^
Time	TG	0.01	0.07	0.76
	HDL-C	0.31	0.01	0.17
	Non-HDL-C	< 0.001	0.12	0.94
Time * Age	TG	0.03	0.05	0.60
	HDL-C	0.06	0.04	0.48
	Non-HDL-C	< 0.001	0.13	0.95
Time * BMI percentiles	TG	0.33	0.01	0.16
	HDL-C	0.33	0.01	0.16
	Non-HDL-C	0.49	0.005	0.10
Time * Gender	TG	0.18	0.02	0.26
	HDL-C	0.11	0.03	0.36
	Non-HDL-C	0.28	0.01	0.19
Time * SEL	TG	0.41	0.02	0.20
	HDL-C	0.88	0.003	0.07
	Non-HDL-C	0.90	0.002	0.07
Time * Gender * SEL	TG	0.57	0.012	0.14
	HDL-C	0.81	0.005	0.08
	Non-HDL-C	0.99	< 0.001	0.05

^a^Computed using alpha =0.05

## Discussion

In this first longitudinal study of lipid profile involving a Middle-Eastern pediatric population, out of 97 subjects aged 11 to 21 years with a baseline abnormal lipid profile, 46.4% still show a persistent abnormal lipid profile 3 years later, whereas the lipid profile has been normalized in the remaining subjects. Most notable are the significant decreases in TG and non-HDL-C observed in girls while, in boys, the only significant change was a decrease in TG. HDL-C did not change significantly in boys, but increased in girls and approached statistical significance. In addition, these changes were mainly observed in the youngest subjects.

Non-HDL-C is a combined measure of the cholesterol content of all atherogenic apolipoprotein B–containing lipoproteins. We used it in the current study since it is considered as a better atherogenic risk marker during childhood compared to LDL-C [4, 21–23]. In addition, non-HDL-C is not affected by the absence of fasting, making comparison between the fasting and the non-fasting states reliable [22, 24]. In our previous report, prepubertal girls (8-11 years) had higher non-HDL-C levels compared to boys [11]. In addition, and only in girls, an inverse relationship was noted between non-HDL-C and age, while in boys no significant changes in non-HDL-C were observed between age groups. The decrease in non-HDL-C observed in the present study was significant only in girls, confirming our previous cross-sectional study results, while, in boys, this decrease was at the limit of significance. The greater decrease in non-HDL-C in girls with younger age could be explained by the increase in HDL-C levels during puberty under the effect of estrogen [25]. However, the decrease in non-HDL-C in boys, even if non-significant, is unclear; one of the possible explanations could be a change in lifestyle that took place following dietary advices in schools or through medias, despite a non-significant change in BMI in boys. These results are in line with the recent longitudinal study of Eissa et al. [18], where throughout puberty, a decrease in non-HDL-C was observed with no significant difference in the pattern of change between males and females, even if in the current study a much more pronounced effect was noted in girls. Both studies are longitudinal, and the improvement in lipid profile is compatible with the American NHANES study which had found an improvement in lipid profile after a 10-year follow-up [26].

We found a significant decrease in TG in both girls and boys with time. In our previous report [11], TG values were higher in older boys while values were lower in older girls. In addition, results from the US NHANES cross-sectional study [14] showed preadolescent peaks of TG in girls. Conversely, Armstrong et al. found a positive correlation between TG and age in girls between 11 and 16 years [15], while the longitudinal study of Eissa et al. showed a progressive increase of TG during puberty only in boys [18]. In our study, the absence of elevation of TG with time in boys could be explained by the fact that the first sampling was taken from a non-fasting population. However, according to Steiner et al. [24], it seems that only 4% of children classified with normal TG when fasting would have elevated postprandial values. Since the observed decrease in TG levels in the current study is much more important, we assume that the fasting effect alone does not explain the drop in TG values. In addition, the effect of a change in BMI could not explain these time changes in lipid parameters since BMI did not change significantly between the two tested periods. Finally, none of the included subjects reported a change in diet and lifestyle at the time of the second sampling, most of them reporting the absence of advices given by the school physician. It is possible though, that the recruitment of boys in our current study was done after they had completed puberty and that this rather late recruitment could have hindered an increase in TG which is normally observed during puberty.

Non-significant changes with time in HDL-C were observed in boys as well as in girls. In our previous report [11], an inverse significant relationship between HDL-C and age was observed in boys while the opposite was observed in girls. Prepubertal girls aged 8-11 have lower HDL-C compared to boys of the same age, with a reversal of this profile at the age of 15-18 years. These results are also observed in the NHANES study [14]. Similarly, Armstrong et al. found a negative correlation between HDL-C and age in boys [15]. On the other hand, in the longitudinal study of Eissa et al. [18], a decrease in HDL-C levels in boys during puberty was noted, whereas, in girls, HDL-C levels did not change but remained greater than HDL-C levels in boys during all pubertal stages [18]. In our study, a non-significant increase in HDL-C levels in girls was observed, while HDL-C did not change in boys. In girls, this result can be explained by the effect of estrogen on HDL-C [25, 27]. However, the absence of decrease in HDL-C levels in boys could be related to the fact that some of them had already begun puberty at the time of the initial study.

Finally, few studies looked at the impact of the socio-economic level (SEL) on lipid variations. In a Turkish study, children from higher SEL have higher TC levels than children from lower socio-economic background [28]. In the United Kingdom, another recent study on an adult population, found higher TG and LDL-C levels in participants from lower SEL; these changes were influenced by obesity, physical activity, and diet [29]. In our previous report study [11], children from lower SEL showed higher TG levels compared to children from higher SEL; our current results found no significant changes in lipid profile according to SEL, suggesting that the improvement in lipid profile is observed irrespective of SEL.

The current study has some limitations. First, the sample size is small; however, it is representative of the overall baseline sample, since the characteristics of the 97 subjects included in the present study were comparable to the baseline characteristics of the 339 subjects with abnormal lipid profile. Second, the impact of a slight change in diet/lifestyle at the time of the second sampling cannot be completely excluded even if all the included subjects assured no changes in their diet/lifestyle. Finally, sampling was taken after a twelve hours of fasting, while sampling in the initial cohort was performed on non-fasting subjects. However, fasting seems to have a very small effect on TGs variation (< 4%) with a negligible effect on HDL-C and non-HDL-C levels [24], while the observed decrease in TG in our study is much higher.

## Conclusion

This is the first study which looks at the longitudinal changes of lipid profile in a pediatric middle-eastern population and analyzes the effect of gender, BMI and SEL. Lipid parameters improved after a mean of 3-year follow up, mainly in girls, promoting a more conservative policy in screening and treating lipid disorders in children. Adopting dietary and lifestyle changes should remain the cornerstone in the primary management of dyslipidemia at an early age.

## Abbreviations

BMI: Body Mass Index
HDL-C: High Density Lipoprotein cholesterol
Non-HDL-C: Non-High Density Lipoprotein cholesterol
SEL: Socioeconomic level
TC: Total cholesterol
TG: triglycerides
